# Rapid Brain MRI Use in a Pediatric Emergency Department

**DOI:** 10.15844/pedneurbriefs-34-21

**Published:** 2020-12-18

**Authors:** Gurpreet Khaira, Jonathan E. Kurz

**Affiliations:** 1 Davee Pediatric Neurocritical Care Program, Division of Neurology, Ann & Robert H. Lurie Children's Hospital of Chicago, Chicago, IL

**Keywords:** Rapid MRI, Emergency Medicine, Neuroimaging, Pediatrics

## Abstract

Investigators from the University of Pittsburgh (Department of Emergency Medicine and Division of Pediatric Radiology) and Feinberg School of Medicine (Division of Emergency Medicine) studied the rates of neuroimaging (rapid brain MRI [rMRI], head CT [HCT], and full MRI) before and after implementation of four rapid MRI protocols in their ED.

Investigators from the University of Pittsburgh (Department of Emergency Medicine and Division of Pediatric Radiology) and Feinberg School of Medicine (Division of Emergency Medicine) studied the rates of neuroimaging (rapid brain MRI [rMRI], head CT [HCT], and full MRI) before and after implementation of four rapid MRI protocols in their ED. Their rationale for this study is that rMRI is a safe, efficient alternative to head CTs. They evaluated differences in time to index neuroimaging, the total length of stay in the ED, rates of unsuccessful index imaging, follow-up imaging, and undetected pathology on the index imaging (for those who had rMRI or HCT as the initial study that was followed by a full MRI within 14 days). The data were retrospectively collected from a high-volume freestanding children's hospital ED. Comparing the control and rMRI periods, rates of rMRI for index imaging were 10.8% and 38.5%, respectively. When looking at HCT use, the rates were 70.0% and 48.5%, respectively. Both differences reached statistical significance. Notably, time to neuroimaging and length of stay in the ED was longer for rMRI versus HCT (182 [IQR 138-255] vs. 86 [IQR 52-137] minutes and 396 [IQR 304-484] vs. 257 [IQR 196-344 minutes respectively). Of note, 3.6% of rMRI studies were unsuccessful versus no HCT studies. No other undetected pathologies were demonstrated in follow-up studies after rMRI, whereas the false-negative rate for HCT was as high as 25%. The authors suggested that rMRI could be seen as a viable alternative to HCT for nontraumatic presentations to the ED. The authors suggested that a longer time to neuroimaging for rMRI may be worthwhile to receive a definitive test if the patient's stability allows. [1]

COMMENTARY. Traditionally, the HCT has been the imaging modality of choice in EDs due to their speed of attainment and thus a minimal need for sedation – the main drawback being, however, that the child is exposed to ionizing radiation. HCTs remain the study of choice, and appropriately so, for traumatic brain injury and intracranial hemorrhage – where emergent interventions, without the need for detailed delineation of injury, are life-saving [2]. In these situations, an rMRI would delay care due to the potential contraindications to being in the MRI suite (e.g., penetrating injury) and the need for readying tubing and machinery to be MRI compatible. Alternatively, MRI brain is superior to HCT for identification of acute stroke, posterior fossa lesions, and in patients with undifferentiated encephalopathy.

Interestingly, there is an emerging evidence-base suggesting that rMRI is at least non-inferior for many other novel modalities. As such, rMRI is emerging as the imaging modality of choice for several different indications – most commonly evaluating cerebral ventricles, as noted by a recent North American survey of pediatric neurosurgeons [3]. It is important to emphasize that a significant reason why rMRI protocols have become so popular is that the sequences have been selected to offer the highest yield for the shortest amount of time in the scanner – such as in rMRI for ventriculomegaly, but also, as the authors utilized, abusive head trauma, stroke, and "neurologic." Consequently, MRI use in pediatric EDs is increasing for several different indications [4]. The authors contributed to this body of research by showing that implementation of rMRI protocols – precisely more than one protocol – leads to reductions in HCT use (and thus exposure to ionizing radiation) without missed diagnoses or increases in the need for follow-up imaging. Rapid MRI protocols should be considered the initial choice for index imaging as access and appropriate protocols.

## Disclosures

The authors have declared that no competing interests exist.
